# Antidemocratic and Exclusionary Practices: COVID-19 and the Continuum of Violence

**DOI:** 10.1017/S1743923X2000046X

**Published:** 2020-07-09

**Authors:** Summer Forester, Cheryl O'Brien

**Affiliations:** 1 Carleton College; 2 San Diego State University

**Keywords:** Intersectionality, violence, gender, feminist security studies, militarization

## Abstract

The global coronavirus pandemic has reified divisions, inequity, and injustices rooted in systems of domination such as racism, sexism, neoliberal capitalism, and ableism. Feminist scholars have theorized these interlocking systems of domination as the “continuum of violence.” Building on this scholarship, we conceptualize the U.S. response to and the consequences of the COVID-19 pandemic as reflective of the continuum of violence. We argue that crises like pandemics expose the antidemocratic and exclusionary practices inherent in this continuum, which is especially racialized and gendered. To support our argument, we provide empirical evidence of the continuum of violence in relation to COVID-19 vis-à-vis the interrelated issues of militarization and what feminists call “everyday security,” such as public health and gender-based violence. The continuum of violence contributes theoretically and practically to our understanding of how violence that the pandemic illuminates is embedded in broader systems of domination and exclusion.

As states started introducing plans to contain the COVID-19 pandemic in early 2020, feminist activists and scholars, among others, began sounding the alarm about the deleterious effects that the pandemic would have on women, communities of color, and other oppressed groups (see, e.g., Enloe 2020; UN Women 2020). These warnings proved prescient: during the last few weeks of March, calls to a Vancouver domestic violence hotline increased by 300% (Daya and Azpiri 2020). In the United States, Native American and Black communities face the highest hospitalization rates for COVID-19 (CDC 2020). Simply put, the pandemic has intensified divisions, inequity, and injustices rooted in systems of domination such as racism, sexism, neoliberal capitalism, and ableism.

These interlocking systems of domination characterize what feminist scholars call the “continuum of violence” (Cockburn 2004, 2012; Kelly 1988; Moser 2001; Wibben 2020). The continuum of violence alerts us to how different manifestations of violence, from the international arena to the most intimate spaces, are interconnected. It lays bare the futility of drawing stark demarcations between peace and war, security and insecurity.

We conceptualize the U.S. response to and the consequences of the COVID-19 pandemic as reflective of the continuum of violence. We argue that crises like pandemics expose the antidemocratic and exclusionary practices inherent in this continuum, which is especially racialized and gendered. We provide empirical evidence of the continuum of violence in relation to COVID-19 vis-à-vis the interrelated issues of militarization and what feminists call “everyday security,” such as public health. The continuum of violence contributes theoretically and practically to our understanding of how the violence that the pandemic illuminates is embedded in broader systems of domination and exclusion. During and beyond this pandemic, deconstructing the continuum of violence demands systemic changes to cultivate more just and democratic societies.

## THE CONTINUUM OF VIOLENCE

Feminist antiwar activists were among the first to highlight different forms of violence, from wife battering to nuclear war, as interconnected and existing on a continuum (Cockburn 2012). This continuum of violence blurs the stark divisions between war and peace, between conflict abroad and militarization at home, and it emphasizes how systemic power differentials are *productive forces* behind myriad forms of violence (Cockburn 2004; Enloe 2007).

While much feminist research on the continuum of violence centers on women's experiences, it recognizes that gender(ed) experiences are not universal and that experiences are shaped and affected by multiple intersecting identities (Cockburn 2012; Collins 2001; Crenshaw 1991; Wibben 2020). As such, an intersectional understanding of the continuum of violence requires us to pay attention to *systems* and the violence that they produce, and how these systems are mutually reinforcing and engender forms of violence that differentially affect vulnerable communities (True 2012; Wibben 2020).

In short, the continuum of violence shows us how structures of violence that dramatically limit people's lives and potential produce a continuity of effects, not episodic cases. Taking these lessons seriously, we contend that the violence being witnessed as the pandemic ebbs and flows cannot be divorced from broader systems of violence and their intersecting manifestations of oppression and power.

## MILITARIZING COVID-19

Militarism and militarization are functions of the continuum of violence. They reinforce and impart martial values such as domination, hierarchical chains of command, and an obsession with force and firepower into civilian spaces (Cohn 2013; Enloe 2007), and “a militarized society is necessarily undemocratic” (Cockburn 2004, 31). As such, militarizing COVID-19 requires an antidemocratic, authoritarian logic and approach to addressing the myriad consequences of the pandemic.

Although feminists have warned against treating COVID-19 like a war (Enloe 2020), the U.S. response to the coronavirus, in both rhetoric and action, has indeed been militarized. For instance, in May 2020, President Donald Trump said “I view the invisible enemy [coronavirus] as a war,” and he called the virus an attack worse than the bombing of Pearl Harbor and 9/11 (BBC News 2020). Meanwhile, Minnesota's governor created a “battle plan” for contending with COVID-19 in long-term care facilities and called in the National Guard to conduct testing (Minnesota Public Radio 2020).

Militaristic responses to COVID-19 purportedly enhance national security. In practice, however, militarism engenders greater *insecurities*, and these are pronounced among intersectionally marginalized communities (Parashar 2018; Rogers 2020). One example of how militarization differentially affects dominant and marginalized groups is the reaction to protests in the United States during the pandemic. In Michigan, Minnesota, Texas, and elsewhere, protests to “open up the economy” were arguably violent, given that some protesters brandished guns. Yet Trump tweeted multiple encouraging messages, such as “LIBERATE VIRGINIA, and save your great 2nd Amendment. It is under siege!”^1^ These protesters were predominantly white, and many of their messages equated quarantine with tyranny, while reopening the economy signaled freedom and patriotism (Jeffrey 2020). Notably, we did not witness police violence in response to these protests.

Surges in activism supporting the Black Lives Matter (BLM) movement have been met, however, with breathtaking brutality and repression by state and federal police officers (Dewan and Baker 2020). Since May 26, Amnesty International (2020) has documented 125 incidents of police violence aimed at antiracism protesters in 40 states. In contrast to the “open up the economy” protests, Trump has referred to BLM protesters as thugs and terrorists, and he threatened to send in troops to “dominate” and suppress these more racially diverse protests (Wise 2020). Hypermilitarized responses to BLM protesters—but *not* to armed protesters demanding states “reopen the economy” despite ongoing coronavirus concerns—speaks to how racism and militarism are mutually constitutive and violent toward nondominant groups (see Rogers 2020).

Exploring the fallout from militarizing COVID-19 exposes the inconsistencies, tensions, and power imbalances inherent to a militarized response. Rhetoric of patriotism and battle-ready strategies chafe against weak and underdeveloped policy responses and the haphazard enforcement of lockdown requirements. Moreover, militaristic language is used strategically and selectively, and always in ways that bolster dominant groups: instead of a “war on racism,” BLM protests are framed as a “war on cops.”

## EVERYDAY SECURITY

The continuum of violence is not only reflected during the pandemic in the racist militarization in the United States, it is also highlighted in what feminists call “everyday security.” Everyday security rejects the idea that states should be the central security concern and instead centers on the well-being of individuals and communities *within* states (Wibben 2020).

A continuum of violence and its systemic racism is thus evidenced by undemocratic health outcomes during the pandemic. In the United States, in comparison with their white counterparts, people of color—especially black people—have experienced disproportionately high rates of COVID-19 infections, hospitalizations, and deaths, and they continue to suffer disproportionately from this pandemic's economic fallout (CDC 2020; Rogers 2020). While race-based discrimination in health care has existed for decades (Cunningham et al. 2017; Mays, Cochran, and Barnes 2007), the pandemic exacerbates these ongoing disparities. Similar to people of color in the United States, indigenous populations worldwide experience exclusion from public health care during and beyond this coronavirus pandemic (Curtice and Choo 2020).

Like racism, gendered oppressions, such as patriarchal control over women's bodies, showcase the continuum of violence and its exclusionary, antidemocratic practices. The continuum of violence prevents women from controlling their own lives through gender-based violence and a lack of reproductive rights (Wibben 2020). In addition to increased (risks of) domestic violence and other forms of violence against women during the pandemic (Godin 2020; Pfitzner, Fitz-Gibbon, and True 2020), women's reproductive rights have been attacked under the guise of protecting medical personnel from COVID-19 by conservative U.S. governors who “have ordered or supported the cessation of” medication and/or surgical abortion (Bayefsky, Bartz, and Watson 2020). Such rescinding of abortion access in times of health system crises frames women's constitutional rights, autonomy, *and* health care needs as “expendable” (Bayefsky, Bartz, and Watson 2020). During a pandemic, access to abortion remains essential, as access to contraception may be reduced under quarantine, financial hardships increase, and unplanned pregnancies still occur. Considering the interlocking economic, gendered, and racist systems of oppression, poor women of color are especially disadvantaged in public health care.

## CONCLUSION

The COVID-19 pandemic exposes a multidimensional continuum of violence that comprises authoritarian, exclusionary practices, and hierarchical relations that undermine democracy and the everyday security of nondominant groups. We have shown that, despite an emphasis on securing the nation through well-formulated battle plans and war-tested policy maneuvers, the United States has categorically failed to protect vast swaths of the population. Ripple effects of the pandemic—such as increases in domestic violence and food insecurity—are part and parcel of systems of structural *violence*—not just structural inequality—that are predicated on the oppression and subordination of women and people of color.

To dismantle this continuum, we need a multipronged global approach that includes a shift in the culture of policing away from militarization to nonhierarchical community engagement and stronger, more robust democratic policies and processes that invest equitably in education and health care and *actively* ameliorate systemic violence. Feminist contributions on intersectionality and the continuum of violence highlight the antidemocratic forces that threaten our security in many ways and call us to justice, peacebuilding, and inclusion of diverse peoples, across all continents, to combat global crises like pandemics and climate change as well as more intimate types of violence.

## Footnotes

1. Donald Trump (@realDonaldTrump), “LIBERATE VIRGINIA, and save your great 2nd Amendment. It is under siege!,” Twitter, April 17, 2020, https://twitter.com/realDonaldTrump/status/1251169987110330372 (30June2020).
